# Compartment Syndrome Resulting From Improper Intraosseous Cannulation: A Case Report

**DOI:** 10.7759/cureus.50248

**Published:** 2023-12-09

**Authors:** Kishan K Desai, Adam J Mann, Faris Azar, Lawrence Lottenberg, Robert Borrego

**Affiliations:** 1 Department of Surgery, Schmidt College of Medicine, Florida Atlantic University, Boca Raton, USA; 2 Department of Surgery, St. Mary’s Medical Center, West Palm Beach, USA

**Keywords:** pre-hospital intervention, transfusion in trauma, intraosseous infusion, emergent fasciotomy, lower extremity swelling, extravasation injury, acute compartment syndrome of leg, intraosseous access, acute trauma care, acute care surgery and trauma

## Abstract

Obtaining adequate vascular access is imperative for effective resuscitative, therapeutic, and diagnostic interventions. The intraosseous (IO) route is indicated when immediate vascular access is needed, and standard central or peripheral intravenous (IV) access is unattainable or would delay therapy in a critical patient. We present a rare case of improper IO line placement in the right proximal tibia of a 30-year-old female involved in a motor vehicle collision, resulting in extravasation of blood products into the surrounding tissue and development of acute compartment syndrome. Emergency Medical Services was unable to obtain IV access in a timely manner, thus a right proximal tibia 45mm IO line was placed, and a unit of whole blood was given with a high-pressure infusor in the field. At the trauma center, the patient’s right lower extremity was severely tense and edematous with no palpable right lower extremity pulses and no Doppler signals. Computed tomography revealed the IO catheter extending through both the proximal and distal cortices of the right tibia. Medial and lateral fasciotomy of the right lower extremity was performed in which all four compartments of the right lower leg were released and a significant hematoma was evacuated from the superficial posterior compartment. This case highlights the importance of IO access as a life-saving intervention while also underscoring the need to educate and familiarize pre-hospital and hospital healthcare personnel in delivering IO access so as to mitigate risks and improve outcomes for critically ill patients.

## Introduction

Obtaining adequate vascular access is a pillar of modern medical therapy. Reliable vascular access is critical to ensure administration of medications and resuscitative fluids, laboratory testing, and contrast for diagnostic procedures [1,2]. In the setting of medical emergencies, intraosseous (IO) access and intravenous (IV) access refer to methods of administering therapies and laboratory tests to patients. IO cannulation involves placing a specialized hollow bore needle through the cortex of a bone into the medullary space wherein a network of microvasculature, medullary sinusoids, and drains to systemic venous vasculature. The most common sites for IO access are the humeral head and proximal tibia; other potential sites include the sternum, clavicle, iliac crest, distal femur, distal tibia, and calcaneus [2].

In the hospital setting, the IO route is indicated when immediate vascular access is needed and standard central or peripheral venous access is unattainable or would delay therapy in a critical patient [3,4]. Similarly, in the pre-hospital setting, IO access can be attained by Emergency Medical Services (EMS) in critical scenarios wherein peripheral venous access is unattainable, and first responders may have limited technical experience. Though rare, known complications of IO access include extravasation, bone fractures, infiltration of medications, development of infection, soft-tissue necrosis, osteomyelitis, or fat embolism [4-7]. We present a rare case of improper IO line placement in the right proximal tibia of a 30-year-old female, resulting in extravasation of blood products into the surrounding tissue and development of compartment syndrome.

## Case presentation

The patient is a 30-year-old female who presented to the trauma center after being involved in a motor vehicle collision. The patient was ejected from the vehicle. EMS was unable to obtain IV access in a timely manner, and a right proximal tibia 45mm IO line was placed in the field. She was hypotensive at the scene and during transport, thus a unit of whole blood was given with a high-pressure infusor through the right tibia IO line on route. Upon arrival at the trauma bay, the patient was severely agitated with waxing and waning mentation. Peripheral IV access was obtained, and the patient underwent rapid sequence intubation for airway protection. She remained hypotensive in the trauma bay with a systolic blood pressure of 54mmHg. Bilateral finger thoracostomies were performed and bilateral chest tubes were placed. A central venous catheter and arterial line were placed. On physical examination, the patient’s right lower extremity had the IO catheter placed by EMS and was severely tense and edematous. She had no palpable right lower extremity pulses with no Doppler signals. She also had a left ear avulsion injury and severe abrasions and lacerations in a grid-type pattern on her left shoulder posteriorly, and additional superficial abrasions on her lower back and buttocks. The patient stabilized with the administration of two units of packed red blood cells.

**Figure 1:**
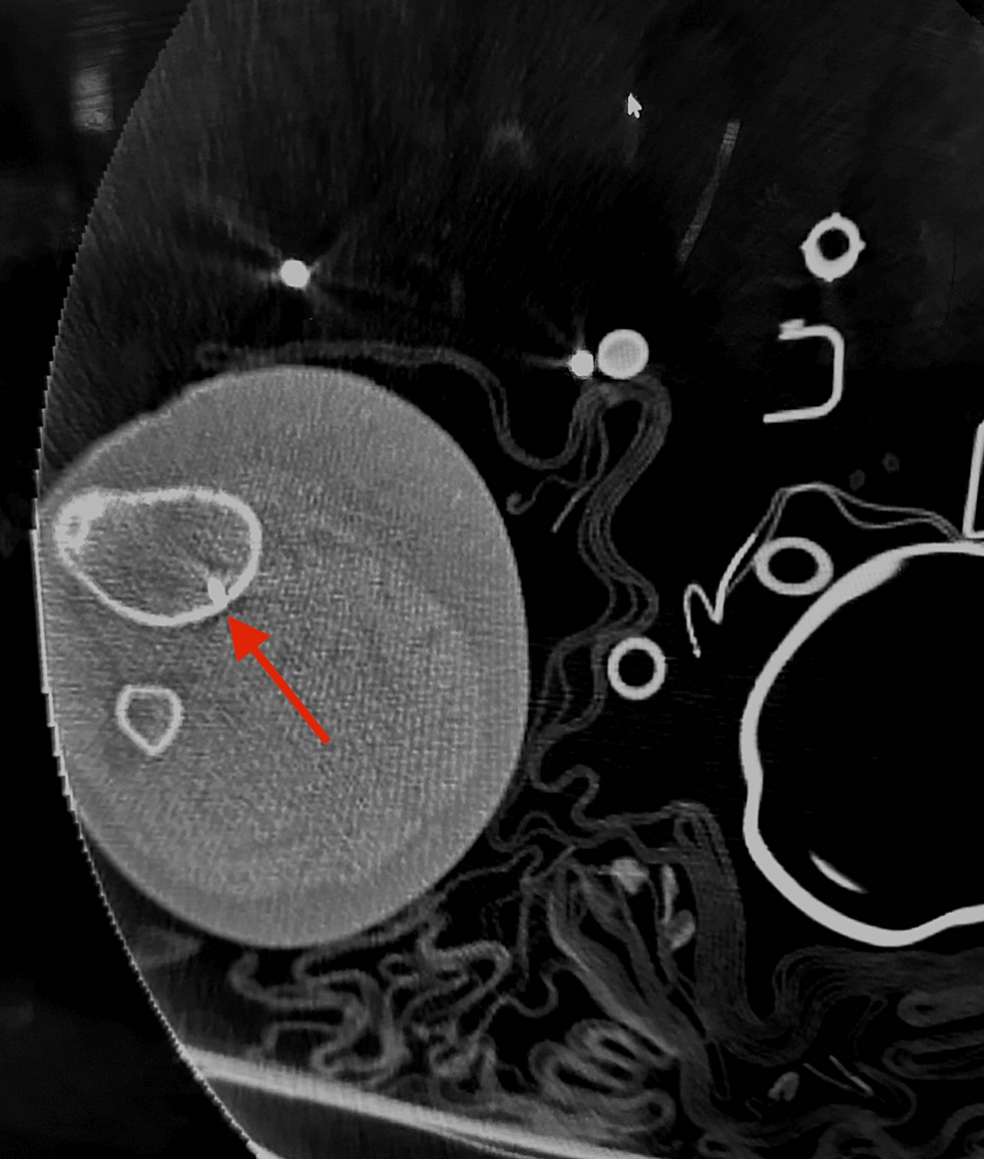
An IO catheter is seen extending into the proximal tibial diaphysis with a tip protruding through the posterior cortex (arrow) on CT of the right lower extremity. IO: Intraosseous

Computed tomography (CT) of the spine revealed mild compression fractures of the lumbar spine at L1 and superior endplate of T8 and T7, and transverse process fractures on the right at L4 and L5. CT of the right lower extremity revealed an IO catheter extending into the proximal tibial diaphysis with the tip protruding through the posterior cortex along with a few foci of gas within the deep musculature posterior to the tibia and knee joint effusion (Figure 1). Due to the physical examination of the right distal lower extremity and the concern for acute compartment syndrome, the IO catheter was removed, and the patient was immediately taken to the operating room for a right distal lower extremity medial and lateral fasciotomy and debridement and washout of her lacerations and abrasions.

Medial and lateral fasciotomy of the right lower extremity was performed under general anesthesia. All four compartments of the right lower leg were released. There was significantly elevated pressure within the superficial posterior compartment and a significant hematoma was evacuated; however, the muscle appeared viable. The wound was packed with surgical gauze and the entire leg was wrapped with bandage rolls. The left upper back was debrided and irrigated with normal saline and a wet to dry dressing was placed over her left upper back and left ear. At the end of the surgery, the patient was hemodynamically stable. She remained intubated and was admitted to the intensive care unit (ICU).

The patient was extubated and transferred out of the ICU after three days. Her upper back wound became infected with purulent drainage, and she received empiric antibiotic treatment with doxycycline and piperacillin-tazobactam. The remainder of her hospital course included hyperbaric oxygen treatment indicated in compartment syndrome to aid with delivery of higher oxygen concentrations to ischemic tissues, decrease surrounding edema at her injury sites, and promote bacteriostatic properties; and multiple visits to the operating room for washout and debridement of her back; primary closure of the right lower extremity wounds; and partial amputation and repair of her left ear. Patient’s multiple mild compression fractures of the thoracolumbar spine were determined to be stable requiring no bracing. The patient worked with Physical and Occupational Therapy extensively and increasingly restored motor and sensory function throughout her stay. The patient was discharged to an acute rehabilitation facility on hospital day 16 for physical and occupational therapy support and continual wound care.

While in acute rehabilitation, she returned to the OR for additional washout and debridement of her back and eventual split-thickness skin graft placement of her back and buttock wounds. To summarize the patient’s hospital stay, she required three days in the ICU receiving mechanical ventilation, a 16-day hospital stay, and 13 visits to the operating room for debridements and eventual split-thickness skin grafting, inpatient acute rehabilitation, and aggressive daily wound care.

## Discussion

Gaining vascular access is of critical importance in resuscitative, therapeutic, and diagnostic measures [8,9]. IO cannulation is the first-line alternative technique in gaining vascular access in critically ill patients when central or peripheral venous access is unattainable in both hospital and pre-hospital conditions. In critically unstable trauma patients without blood pressure, IO success rates are twice as high as IV-line placement [2]. Nevertheless, IO line placement is not without risk or complication including, but not limited to extravasation and compartment syndrome. Compartment syndrome is a condition in which pressure either due to fluid, such as blood, or swelling builds to perilous levels within an osteofascial compartment of the body. This buildup of pressure within a relatively constrained compartment can threaten circulation and oxygenation of tissue, and nerve function. Common indications of compartment syndrome include pain, pallor, paresthesia, pulselessness, and paralysis. If pressure is not relieved emergently in the threatened compartment, cellular and functional damage can result [10]. Procedurally, this relief of pressure is achieved by fasciotomy. 

Proper placement of an IO line is dependent on the procedure setting, patient’s condition, anatomy, weight, and provider’s clinical judgment. Equally so, deviation from this protocol can contribute to an increased risk of complication. For proximal tibial placement, the IO line insertion site is approximately 2cm medial to the tibial tuberosity along the flat aspect of the tibia on an extended leg; the needle is subsequently aimed at a 90-degree angle to the bone for insertion with depth of insertion gauged by markings on the needle itself [11]. Ideally, insertion sites necessitate a flat insertion surface, thin cortical bone, an adequately large medullary cavity, and easy anatomical landmarks so as to avoid misplacement [5]. 

**Figure 2:**
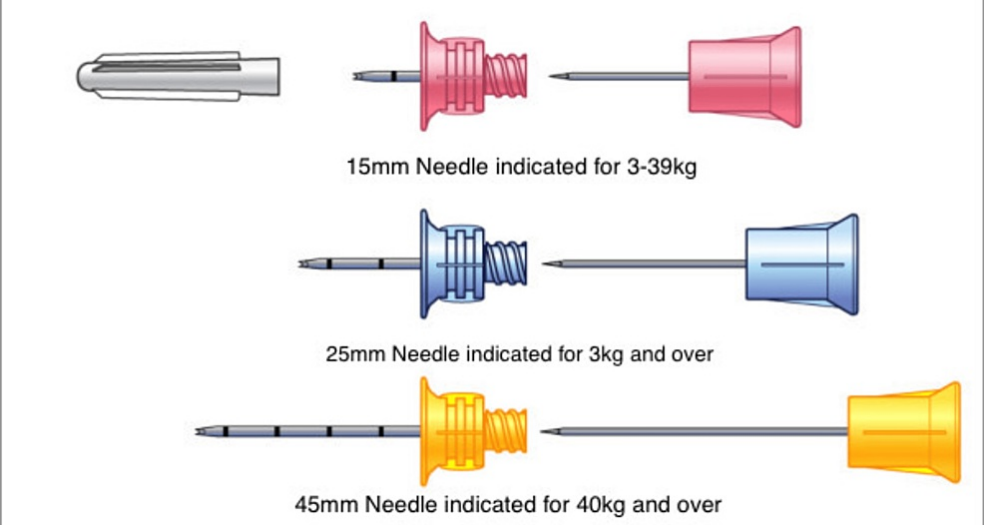
Current day IO vascular access systems utilize three needle sizes indicated for single patient use: 15mm long needles for 3-39 kg patients, 25mm long needles for 3 kg and heavier patients, and 45mm needles considered at the proximal humerus site in 40kg and heavier patients, or patients with excess tissue/edema over any insertion site. Source: [12] IO: Intraosseous

Current day IO vascular access systems operate most commonly with battery-powered insertion with three needle sizes indicated for single patient use: 15mm long needles for 3-39kg patients, 25mm long needles for 3kg and heavier patients, and 45mm needles. The 45mm needle should be considered at the proximal humerus site in 40kg and heavier patients, or patients with excess tissue/edema over any insertion site (Figure 2) [11]. The patient in this case was an obese female with a weight of 104.5kg. Per the guidelines listed above, either a 25mm or a 45mm needle would be appropriate; however, the 45mm IO catheter went through both the proximal and distal cortices of the right tibia. Improper needle size can result in incomplete penetration of the needle through the cortex or disruption of the distal bone cortex and can result in extravasation of fluids.

Typical indications for determining proper placement and insertion of the IO needle include loss of resistance or “pop” on entering the marrow cavity, the needle remaining upright upon insertion without support, both the easy administration of fluids and aspiration of blood/bone marrow without resistance and the administration of 2mL of saline without subcutaneous tissue swelling [11,13]. The IO line is secured to avoid displacement in transport, or during care. Best practices suggest rapid vascular infusion through the IO line under a pressure of 300mmHg so as to maximize flow [11,14]. Extravasation of fluids has been associated with the infusion of fluids under pressure; however, a review of the literature reports no significant differences in extravasation rates between gravity and 300mmHg pressure infusions under the condition of proper and uncomplicated needle placement [15].

In our case, the improper placement of an IO line in the right tibia in the prehospital setting and subsequent administration of one unit of whole blood resulted in extravasation and compartment syndrome of the right lower extremity. The IO catheter went through both the proximal and distal cortices of the right tibia instead of being placed and secured in the medullary space for a non-collapsible entry point into the systemic venous system. This placement resulted in subsequent extravasation of administered blood products into the osteofascial space. Notably, the incidence of extravasation of medications administered through IO access is 3.7% and development of compartment syndrome secondary to IO placement is even lower at 0.6% [16]. A systematic review and meta-analysis evaluating the efficacy and efficiency of IO access compared to IV access for trauma resuscitation in pre-hospital care indicated that the success rate on the first attempt of IO access was much higher than that of IV access for trauma patients, and the mean procedure time was significantly reduced. The findings of this review suggest that IO access should be considered for hypotensive trauma patients, especially those who are under severe hypovolemic shock [4]. Multiple studies show high success rates by physicians, nurses, and paramedics in performing IO cannulation of adults and pediatric patients [7,8]. In fact, studies have shown there is no statistically significant difference in complications between the IO and IV interventions [4]. 

IO access remains a trusted and evidence-based method for gaining vascular access when standard peripheral IV access is not possible. Certainly, there are risks and potential complications that can arise from IO cannulation; however, proper training in timely IO access is imperative to mitigating risks and improving morbidity and mortality. Furthermore, technological and mechanical safeguards can be investigated and placed into standard practice to reduce complications. This may include incorporating the use of Point-of-Care Ultrasound with Color Doppler to visualize and confirm proper placement and flow functionality of the IO line or development of IO drills pressure sensitive to cortical and medullary bone density [17,18]. Thus, educating both pre-hospital and hospital healthcare team members in IO placement techniques and incorporating technological and mechanical safeguards for IO access can decrease the incidence of complications [19,20].

## Conclusions

Obtaining adequate vascular access is imperative for effective resuscitative, therapeutic, and diagnostic interventions. Vascular access by IO or IV cannulation allows for administration of medical therapeutics, and laboratory testing. While IV cannulation is the standard for gaining vascular access, IO cannulation is the first-line alternative technique in gaining vascular access in critically ill patients wherein central or peripheral venous access is unattainable or may be delayed.

Complications of IO access can include extravasation, bone fractures, infection, abscess formation, or embolic development. Complications are rare and statistically insignificant when compared to IV access. To avoid complications, we recommend in adults IO needles be placed in the humerus for rapid infusion of blood rather than the tibia if there are no contraindications. Educating and familiarizing pre-hospital and hospital healthcare personnel on the efficacy and techniques of IO access and developing technological and mechanical safeguards can increase competency in delivering this life-saving intervention, mitigate risks, and improve outcomes for critically ill patients.
